# Navigating the gut–metabolite–immune axis: enhancing efficacy and mitigating toxicity of immune checkpoint inhibitors

**DOI:** 10.3389/fimmu.2026.1803970

**Published:** 2026-07-13

**Authors:** Yu Zhang, Shengnan Wang, Shuang Chang, Yuanyuan Li, Yexing Dang, Zhihao Wang

**Affiliations:** Department of Geriatrics, The First Hospital of Jilin University, Changchun, China

**Keywords:** cancer therapy, gut microbial metabolites, gut microbiome, immune checkpoint inhibitors, metabolism

## Abstract

Immune checkpoint inhibitors (ICIs) have revolutionized the oncological landscape by disrupting inhibitory pathways, notably programmed cell death protein-1/programmed death-ligand 1 (PD-1/PD-L1) and cytotoxic T-lymphocyte-associated antigen-4 (CTLA-4) pathways, thereby reinvigorating host antitumor immunity. Although these agents have emerged as frontline standard therapies for malignancies, their clinical utility remains limited. Interpatient therapeutic variability is inextricably linked to the composition and functional capacity of the gut microbiome. The underlying mechanisms appear to involve a complex dialogue between the microbiota and host immune system, where microbial metabolites serve as critical mediators in remodeling the tumor microenvironment. Despite these insights, progression in the field remains constrained due to heterogeneity in study cohorts and sample-processing methodologies, hindering the establishment of reproducible individualized predictive models and clinical intervention strategies. Consequently, there is an urgent need to systematically delineate the microbiome–metabolite–immune axis to optimize the balance between ICI efficacy and systemic toxicity. By synthesizing the latest evidence, this review aimed to highlight the pivotal roles of specific taxa, including *Bacteroides*, *Bifidobacterium*, and *Akkermansia muciniphila*, in ICI efficacy. These microbes and their metabolic byproducts potentiate therapeutic responses by enhancing dendritic cell cross-presentation and promoting CD^8+^ T-cell infiltration, often via activation of the cyclic GMP-AMP synthase-stimulator of interferon genes or nucleotide-binding oligomerization domain-containing protein 2 signaling pathways. Furthermore, these microbial components demonstrate the ability to protect the heart and colon against inflammation and barrier disruption, thereby mitigating immune-related adverse events. Although the feasibility and safety of interventions such as fecal microbiota transplantation and supplementation with next-generation encapsulated probiotics, postbiotics, or dietary fiber have been demonstrated in preclinical and Phase I trials, substantial hurdles remain. Future progress requires large-scale, multicenter, standardized, longitudinal studies integrating metagenomics and metabolomics to construct robust cross-cancer and cross-population predictive models. Such rigorous validation would enable the development of precise microbial interventions that maximize therapeutic gains while minimizing the incidence of adverse reactions.

## 1 Introduction

The landscape of oncological therapeutics has undergone a profound shift over the past 15 years, largely driven by the advent of immune checkpoint inhibitors (ICIs). These agents, which target programmed cell death protein 1 (PD-1), programmed death-ligand 1 (PD-L1), and cytotoxic T-lymphocyte-associated protein 4 (CTLA-4), have fundamentally transformed clinical cancer care (1). The modern era of checkpoint blockade began in 2011 with the approval of the anti-CTLA-4 antibody ipilimumab for advanced melanoma management (2). Over the following decade, the therapeutic arsenal rapidly expanded with the development of additional immune pathway inhibitors—most notably, the PD-1 antagonist nivolumab and the PD-L1 inhibitor atezolizumab (3, 4). Consequently, ICIs have achieved a clinical status comparable to that of established modalities, such as surgery, radiotherapy, and chemotherapy, and have become the first-line standard treatment for multiple malignancies, including non-small cell lung cancer (NSCLC) (5), advanced melanoma (6), and platinum-refractory urothelial carcinoma (7).

Despite these transformative advances, only a subset of patients achieves durable benefit from checkpoint-based immunotherapies in clinical practice, reflecting substantial interpatient heterogeneity. This variable treatment efficacy is thought to arise from the complex interplay between the tumor microenvironment (TME) and the intrinsic immune state of the host (8). Several biomarkers have been proposed to explain this variability, including tumor PD-L1 expression (9), tumor mutational burden (TMB) (10), and the functional activity of the interferon-gamma (IFN-γ) signaling pathway (11).

As the clinical application of ICIs continues to expand, research over the past decade has increasingly focused on the microbiome and its metabolic byproducts as systemic regulators of immune function. Given its capacity to modulate immunity at both local and systemic levels, the gut microbiota composition has emerged as a plausible determinant of therapeutic heterogeneity. This concept has been supported by extensive preclinical evidence demonstrating that the gut microbiome influences the efficacy of tumor immunotherapy (12, 13). Moreover, animal models and early-phase clinical studies have indicated that interventions such as fecal microbiota transplantation (FMT) can enhance immune cell infiltration into the TME, thereby improving response rates to ICI therapy (12, 14). Microbial metabolic output varies widely across gut ecosystems; for example, butyrate, a key short-chain fatty acid (SCFA) produced by the gut bacteria, has been shown to modulate cytotoxic CD^8+^ T-cell function in animal models and potentiate anti-cancer immune responses (11).

Accordingly, systematic characterization of the interactions linking the gut microbiome, its metabolome, and ICI efficacy represents a critical frontier in tumor immunology. Therefore, this review aimed to synthesize the current evidence on the mechanisms by which the gut microbiota and microbe-derived metabolites regulate ICI efficacy and immune-related adverse events (irAEs), and to evaluate emerging microbiome-targeted interventions to improve clinical outcomes.

## 2 Methods

We conducted a comprehensive literature search across PubMed/MEDLINE, Web of Science Core Collection, and Embase from inception to May 1 2026. The search strategy combined three conceptual domains: (1) gut microbiota and specific taxa (*Akkermansia*, *Bacteroides*, *Bifidobacterium*, *Faecalibacterium*, *Lactobacillus*, *Enterococcus*), microbiome-targeted interventions (FMT, probiotics, prebiotics, postbiotics, dietary fiber), and microbial metabolites (SCFAs, indole derivatives, TMAO, tryptophan metabolites); (2) ICIs and their targets (PD-1, PD-L1, CTLA-4, LAG-3, TIM-3, TIGIT) and agents (ipilimumab, nivolumab, pembrolizumab, atezolizumab, relatlimab); and (3) clinical or mechanistic outcomes, including treatment response, resistance, irAEs (colitis, myocarditis, hepatitis, pneumonitis), and signaling pathways (cGAS-STING, NOD2, AhR). MeSH terms and free-text keywords were used in combination. Only English-language full-text articles were finally included. The exact search strings for each database are provided in **Supplementary Material 1**.

Original studies, randomized controlled trials, prospective or retrospective cohort studies, and preclinical mechanistic investigations (*in vivo* or *in vitro*) that explicitly examined how the gut microbiota or microbial metabolites affect ICI efficacy or toxicities were included. High-quality systematic reviews, meta-analyses, and consensus statements or guidelines were also considered. Exclusion criteria comprised: studies examining microbial interventions or metabolites in cancer without explicit ICI relevance (e.g., chemotherapy or surgery only); conference abstracts, editorials, commentaries, and narrative reviews without original data; case reports or series with fewer than three cases and no control comparison; non-human studies on non-cancer models lacking translational relevance to ICI therapy; and articles with unobtainable full text after institutional requests.

Two authors (Z.Y. and W.S.) independently screened titles and abstracts, reviewed full texts of potentially eligible articles, and resolved disagreements through discussion with a senior author (Z.W.) until consensus was reached. A standardized form was used to record first author, year, study design, cancer type, ICI regimen, sample size, microbial taxa or metabolites studied, key findings, mechanistic pathways, and the reported association with ICI efficacy or toxicities. To ensure consistent reporting of microbiome-related methodology across included studies, data extraction was informed by the STORMS (Strengthening The Organization and Reporting of Microbiome Studies) guidelines, particularly regarding sample processing, sequencing platforms, and bioinformatic pipelines. As a narrative review focused on mechanistic synthesis, formal risk-of-bias assessment, quality grading, and statistical synthesis were not performed. The database search was locked in May 1 2026.

## 3 Mechanisms of ICIs

ICIs represent a transformative class of therapeutics designed to disrupt the binding between inhibitory checkpoint proteins and their cognate ligands, thereby reinstating the capacity of the immune system to recognize and eliminate malignant cells (15). At the molecular level, the efficacy of ICIs primarily depends on modulation of the CTLA-4 and PD-1/PD-L1 axes.

### 3.1 Mechanistic basis of CTLA-4 inhibition

CTLA-4, a member of the CD28 immunoglobulin subfamily, is a critical co-inhibitory molecule expressed on the surface of T cells. Its primary function is to antagonize stimulatory signals of CD28 by competing for the shared ligands CD80 and CD86. However, CTLA-4 possesses a significantly higher affinity for these molecules than that of CD28, effectively quenching T-cell activation at its source.CTLA-4 inhibitors, such as ipilimumab, obstruct the interaction with B7 molecules, facilitating robust T-cell activation and proliferation. This blockade removes the “brakes” on effector T cells and impacts regulatory T cells (Tregs), which are indispensable for maintaining self-tolerance. Further evidence suggests that, in addition to blocking inhibitory signals, CTLA-4 may actively deplete Tregs within the TME, further amplifying the systemic antitumor response (16).

### 3.2 The PD-1/PD-L1 axis and therapeutic interruption

PD-1 is an inhibitory receptor broadly expressed across a diverse spectrum of immune cells, including activated T and B cells, natural killer (NK) cells, NKT cells, monocytes, myeloid-derived suppressor cells, and tumor-associated macrophages. PD-1 interacts with two primary ligands: PD-L1 (B7-H1) and PD-L2 (B7-DC). While PD-L2 expression is thought to be restricted to dendritic cells (DCs), macrophages, and bone marrow-derived mast cells, PD-L1 is more ubiquitously expressed, not only in immune and non-lymphoid tissues but also in various malignant cells, making it the predominant focus of current therapeutic strategies (17).Ligation of PD-1 by PD-L1 triggers the activation of SH2-containing protein tyrosine phosphatase 2 (SHP2), which interferes with the downstream PI3K/Akt signaling pathway. This biochemical cascade leads to a marked reduction in interleukin-2 (IL-2) and interferon-gamma (IFN-γ) production, ultimately arresting T-cell proliferation and driving the cell toward a state of anergy or exhaustion.

### 3.3 LAG-3: structure, function, and clinical validation

LAG-3 (CD223) is a type I transmembrane glycoprotein of the immunoglobulin superfamily, expressed on activated CD4^+^ and CD8^+^ T cells, Tregs, and NK cells (18, 19). LAG-3 cooperates with PD-1 to drive T-cell exhaustion, yet their contributions are functionally distinct: PD-1 primarily restrains T-cell proliferation, whereas LAG-3 specifically attenuates T-cell effector functions, such as cytokine secretion and cytotoxicity (20). LAG-3 has been clinically validated in the phase 2–3 RELATIVITY-047 trial (NCT03470922): in previously untreated advanced melanoma, relatlimab plus nivolumab improved progression-free survival (PFS) compared with nivolumab monotherapy (10.1 vs. 4.6 months) (18, 19). These findings prompted the 2022 U.S. Food and Drug Administration’s approval of fixed-dose combination Opdualag for unresectable or metastatic melanoma, establishing LAG-3 as the third clinically validated immune checkpoint target after CTLA-4 and PD-1/PD-L1 (19). Comparable efficacy between nivolumab plus relatlimab and nivolumab plus ipilimumab (36-month PFS rate: 36% vs. 39%) has also been demonstrated, although substantially fewer grade 3–4 treatment-related AEs occurred with nivolumab plus relatlimab (23% vs. 61%), suggesting a superior risk–benefit ratio (21).

### 3.4 TIGIT: from NK cell biology to clinical development

T-cell immunoreceptor with Ig and ITIM domains (TIGIT) is a member of the PVR/nectin family of co-inhibitory receptors expressed on activated CD8^+^ and CD4^+^ T cells, Tregs, NK cells, and innate lymphoid cells (22, 23). TIGIT binds CD155 (PVR) and CD112 (PVRL2) on tumor cells and antigen-presenting cells, competing with the co-stimulatory receptor CD226 (DNAM-1) for shared ligands. With an affinity for CD155 approximately 30- to 100-fold higher than that of CD226, TIGIT effectively outcompetes CD226 for ligand binding and suppresses signal activation (22, 24). A defining feature of TIGIT biology is its dominant role in driving NK cell exhaustion. In tumor-bearing mice and patients with colon cancer, TIGIT—rather than CTLA-4 or PD-1—was identified as the immune checkpoint receptor most tightly linked to NK cell dysfunction (23).

The clinical development of anti-TIGIT antibodies has yielded mixed results. In the phase II CITYSCAPE trial, first-line tiragolumab plus atezolizumab improved the objective response rate over atezolizumab alone in PD-L1–positive NSCLC (37% vs. 21%), with the most pronounced benefit in the high PD-L1 expression subgroup (66% vs. 24%) (25). However, subsequent phase III trials have failed to corroborate these findings: SKYSCRAPER-01 (PD-L1–high NSCLC), SKYSCRAPER-02 (extensive-stage small cell lung cancer), and SKYSCRAPER-06 (non-squamous NSCLC) all missed their primary endpoints, and SKYSCRAPER-06 suggested worse outcomes in the tiragolumab arm (22). The IMbrave152/SKYSCRAPER-14 trial in patients with hepatocellular carcinoma likewise failed to translate early positive signals, leading to the termination of several anti-TIGIT programs, including vibostolimab and ociperlimab (22). These setbacks underscore the context-dependent nature of TIGIT biology and imply that biomarker-driven patient selection—incorporating TIGIT/CD226 expression ratios, tumor-infiltrating NK cell density, or gut microbiome profiles—may be required to identify responsive patients (22, 23).

### 3.5 TIM-3: molecular architecture and immunoregulatory functions

T-cell immunoglobulin and mucin-domain-containing protein 3 (TIM-3, encoded by *HAVCR2*) is a type I transmembrane glycoprotein selectively expressed on terminally differentiated Th1 cells, CD8^+^ cytotoxic T lymphocytes, NK cells, and DCs (26, 27). Clinically, TIM-3 upregulation has emerged as a compensatory resistance mechanism following PD-1 blockade, rendering TIM-3 an “escape” checkpoint exploited by tumors upon pharmacological inhibition of the PD-1 axis (26, 28). This mechanistic rationale underpins the therapeutic potential of dual TIM-3/PD-1 targeting to overcome acquired resistance and magnify antitumor responses.

## 4 Deciphering the Heterogeneity of Microbiome–ICI interactions: lessons from divergent experimental landscapes

The primary checkpoint blockade targets, CTLA-4 and the PD-1/PD-L1 axis, form the cornerstone of modern immunotherapy. CTLA-4 is constitutively expressed on Tregs, where it outcompetes CD28 for B7 ligands to prevent aberrant T-cell activation, whereas PD-L1 binding to PD-1 exerts a negative regulatory effect on T-cell function (29, 30). Within the TME, malignant cells frequently exploit these pathways by upregulating CTLA-4 and PD-L1 to circumvent immunosurveillance (31). While ICIs are designed to reinvigorate exhausted T cells and restore the host antitumor response (32), both clinical and preclinical data have revealed that the gut microbiota significantly modulate therapeutic efficacy, explaining, in part, the marked interpatient variability observed in clinical practice (12).

Ipilimumab, the first successful ICI approved for the treatment of advanced melanoma, provides early insight into this relationship. Clinical trials have revealed that CTLA-4 blockade induces fluctuations in antibody titers against specific intestinal bacteria during the induction phase. However, contrary to the expectation of uniform “immune enhancement,” these titers do not follow a linear upward trend; rather, they exhibit heterogeneous patterns, including increases, decreases, or bidirectional shifts (33). This volatile antibody response contributes to the microbial dysbiosis observed in patients treated with ipilimumab. Furthermore, Vétizou et al. evaluated 25 patients with melanoma and found that CTLA-4 blockade reshaped the microbiota, specifically favoring the growth of *B. fragilis*. Crucially, the antitumor efficacy of the drug was dependent on specific *Bacteroides* species, such as *B. thetaiotaomicron* and *B. fragilis*. In mice, FMT from responders to non-responders recapitulated the clinical phenotypes, firmly linking microbial composition to ICI response (12).

Similar phenomena have been observed in anti-PD-1 therapies. A previous prospective study of 112 patients with metastatic melanoma discovered that responders possessed significantly higher α-diversity and an enrichment of *Ruminococcaceae*, *Faecalibacterium*, and *Clostridiales* compared with non-responders (34). Although FMT in mice transferred the response characteristics, only *Ruminococcaceae* remained significantly enriched in murine models (34). This finding aligns with previous observations (35) that only a fraction of human taxa (approximately 53%) successfully colonize murine models. While confirming the microbiome–ICI link, these studies highlight a significant complexity; whereas Vétizou et al. identified *Bacteroides* as the key driver of anti-CTLA-4 efficacy, Gopalakrishnan et al. pointed toward *Faecalibacterium* and *Ruminococcaceae* as determinants of anti-PD-1 efficacy. This divergence suggests that different ICIs may rely on distinct “optimal” microbial signatures rather than on universally beneficial flora.

Further, a large-scale prospective study by Derosa et al. involving 338 patients with NSCLC examined the role of *Akkermansia muciniphila* (Akk) (36). By stratifying patients into Akk-negative, -low, and -high groups, they found that the Akk-low group achieved the longest median overall survival (OS; 27.2 months), while the Akk-high group fared the worst (7.8 months) (36). These findings suggest that the relative abundance of Akk is a more precise prognostic indicator than the presence of Akk alone and challenge the simplistic binary of “good” versus “bad” bacteria, as an overabundance of a typically beneficial microbe such as Akk was associated with poor outcomes. Furthermore, the groups had distinct taxonomic profiles: the Akk-high group was enriched with *Ruminococcus* and *Alistipes* species, whereas the Akk-low group had an abundance of *Veillonella parvula* and *Actinomyces*. The contradiction between these findings and those of Gopalakrishnan et al.—where *Ruminococcaceae* were deemed beneficial—highlights the cohort-dependent nature of microbial roles, which appear highly sensitive to cancer type, host genetics, and clinical variables.

Intraspecific diversity adds another layer of complexity. Lee et al. demonstrated that among four *Bifidobacterium* strains, only *B. bif_K57* and *B. bif_K18* synergized with PD-1 blockade to reduce tumor burden (37). This finding suggests that probiotic interventions must move beyond broad genus-level supplementation to functionally validate strain-specific therapies. Clinical variables, such as age, also play a role: Sivan et al. found that commensal *Bifidobacterium* bolstered anti-PD-L1 efficacy (38), while another study noted that aged mice showed enhanced responsiveness to anti-PD-1 compared to their younger counterparts, possibly due to an age-associated “enterotype” (39, 40).

Finally, the effect of therapeutic regimens cannot be overlooked. Frankel et al. examined four different ICI protocols and found that while *Bacteroides caccae* appeared to be a broad marker of response, the specific combination of ipilimumab plus nivolumab was associated with *Faecalibacterium prausnitzii*, whereas pembrolizumab monotherapy favored *Dorea formicigenerans (*41). These data confirm that microbial influence is context-dependent, necessitating a tailored discussion for each specific treatment modality.

In summary, despite the wealth of evidence linking the gut microbiome to ICI efficacy, the field lacks a universal and highly reproducible signature. This is likely due to variances in technology, methodology, and sample processing—consistent with the findings of Lee et al. (42) regarding cohort dependency. Moving forward, large-scale metagenomic studies with standardized protocols accounting for clinical covariates and specific cancer subtypes are essential to decipher the intricate influence of human gut microbiota on immunotherapy outcomes. Collectively, these findings underscore the potential of specific microbial signatures as predictive biomarkers for ICI therapy. Beyond Western cohorts, emerging evidence from Asian populations underscores the universal relevance of microbial biomarkers. In a prospective study of 21 Chinese patients with advanced NSCLC receiving anti-PD-1 therapy plus chemotherapy, baseline presence of *Bifidobacterium breve* emerged as an independent prognostic factor for PFS. The *B. breve*-positive group achieved a significantly longer median PFS (not reached vs. 106 days) and higher disease control rate than the *B. breve*-negative group, suggesting that this species may serve as a predictive biomarker in chemotherapy–ICI combination regimens (43).

Complementing observational cohorts, interventional trials have begun to explore whether deliberate modulation of the microbiome can prime patients for successful ICI therapy. Glitza et al. conducted a randomized, placebo-controlled, biomarker-stratified Phase Ib trial in patients with melanoma, evaluating vancomycin preconditioning followed by SER-401—a *Firmicutes*-enriched spore formulation—during anti-PD-1 therapy. Translational analyses demonstrated that preconditioning with vancomycin was associated with gut microbiota disruption and impaired immunity, with incomplete recovery at ICB administration, particularly in patients with high baseline *Ruminococcaceae* levels. These results highlight the double-edged nature of antibiotic preconditioning and provide a cautionary framework for future microbial intervention strategies (44). Key findings regarding the impact of the gut microbiota on ICI efficacy across different cohorts are summarized in **Table 1**.

**Table 1:** Impact of the gut microbiota on the clinical and preclinical efficacy of ICIs.

ICI regimen	Associated taxa	Key findings and mechanistic insights	Study context	References
Anti-CTLA-4 (ipilimumab)	B. fragilis, B. thetaiotaomicron	CTLA-4 blockade promotes the growth of B. fragilis; the antitumor effect of the therapy is fundamentally dependent on these specific species via Th1 responses and DC activation.	Clinical: 25 melanoma patients; *In vivo*: Germ-free C57BL/6 mice reconstituted with patient FMT, MCA-205 sarcoma model	Vetizou et al., 2015 (12)
Anti-PD-1 (pembrolizumab/nivolumab)	Faecalibacterium, Ruminococcaceae, Clostridiales	Responders exhibit significantly higher alpha-diversity; enrichment of Ruminococcaceae and Faecalibacterium correlates with enhanced systemic and antitumor immunity.	Clinical: 112 metastatic melanoma patients; *In vivo*: Germ-free mice with patient FMT, MCA-205 melanoma model	Gopalakrishnan et al., 2018 (34)
Anti-PD-1 (nivolumab/pembrolizumab)	Akkermansia muciniphila	Stratified by relative abundance, the Akk-low group (<4.8%) achieved a superior mOS of 27.2 months compared to 7.8 months in the Akk-high group.	Clinical: 338 advanced NSCLC patients; *In vivo*: BALB/c and C57BL/6 mice, CT26/4T1/B16F10/MCA-205/MC38 syngeneic models	Derosa et al., 2022 (36)
Combination (ipilimumab + nivolumab)	F. prausnitzii, B. thetaiotaomicron, H. filiformis	Responders to combination therapy display a distinct microbial signature divergent from monotherapy.	Clinical: Multiple ICI cohorts (metastatic melanoma); Metagenomic shotgun sequencing and unbiased metabolomic profiling	Frankel et al., 2017 (41)
Anti-PD-L1	Bifidobacterium (B. longum, B. breve, B. adolescentis)	Commensal Bifidobacterium species augment antitumor immunity by facilitating dendritic cell maturation and enhancing therapeutic efficacy.	*In vivo*: C57BL/6 mice, B16-F10 melanoma subcutaneous model; Oral gavage of Bifidobacterium cocktail	Sivan et al., 2015 (38)
Anti-PD-1	Bifidobacterium bifidum (Strains K57/K18)	Efficacy is strain-specific; only certain B. bifidum strains synergize with PD-1 inhibitors to effectively reduce tumor burden via enhanced CD4+/CD8+ T cell activation.	*In vivo*: C57BL/6 mice, MC38 colorectal cancer model and additional syngeneic models	Lee et al., 2021 (37)
Anti-PD-1/Anti-PD-L1	L. rhamnosus GG (LGG)	LGG activates the cGAS-cGAMP-STING axis in dendritic cells to induce IFN-beta production, enhancing cross-priming of CD8+ T cells and tumor infiltration.	*In vivo*: C57BL/6 mice, MC38 colorectal cancer and B16-F10 melanoma; *In vitro*: GM-CSF/FLT3L-derived BMDCs	Si et al., 2022 (80)
Anti-PD-1 + FMT (pembrolizumab/nivolumab)	Donor-derived microbiota (loss of Enterocloster, Streptococcus, Clostridium spp. post-FMT)	FMT from healthy donors prior to anti-PD-1 achieved ORR 80% in NSCLC and 75% in melanoma (ipilimumab+nivolumab); loss of deleterious baseline species mediates response.	Clinical: Phase 2 trial; 29 patients (15 treatment-naive NSCLC with PD-L1>=50%, 14 melanoma)	Duttagupta et al. (FMT-LUMINate), 2026 (92)
Anti-PD-1 (nivolumab) + FMT	Donor-derived immunogenic microbes (Bacteroides stercoris, Prevotella spp., SCFA producers)	FMT+nivolumab in ICI-refractory MSS gastric cancer achieved ORR 20% and DCR 40%; validated microbial signatures for PD-1 responsiveness in independent cohort.	Clinical: Phase I; 10 patients (8 gastric, 2 colorectal) refractory to anti-PD-(L)1; NCT04130763	Zhang et al., 2026 (93)
Cabozantinib + nivolumab + Live bacterial supplementation	Multi-strain live bacterial consortium (probiotic supplementation)	Randomized Phase 1 trial demonstrating live bacterial supplementation modulates gut microbiota and enhances ICI efficacy in combination with cabozantinib/nivolumab.	Clinical: Randomized Phase 1; Metastatic renal cell carcinoma (mRCC) patients	Ebrahimi et al., 2024 (88)
Anti-PD-1 + Chemotherapy	Bifidobacterium breve	Presence of B. breve is an independent prognostic factor for PFS; B. breve+ group had significantly longer mPFS (NR vs 106 days) and higher DCR (90.0% vs 12.5%).	Clinical: Prospective cohort; 21 Chinese advanced NSCLC patients receiving anti-PD-1 plus chemotherapy	Zhao et al., 2023 (43)
Anti-PD-1 (neoadjuvant/adjuvant)	Microbiome modulation post-antibiotic preconditioning	Randomized Phase Ib trial evaluating antibiotic preconditioning impact on microbiome and immunity in melanoma patients receiving ICIs; biomarker-stratified design.	Clinical: Randomized Phase Ib; Melanoma patients with antibiotic preconditioning vs control	Glitza et al., 2024 (44)
Anti-PD-1	Bacteroides fragilis BF839	BF839 inhibits tumor growth via cGAS-STING signaling and synergizes with anti-PD-1 to induce regression; increases CD8+ T cell infiltration in TME.	*In vivo*: C57BL/6 mice with B16-F10 melanoma; *In vitro*: B16-STING-KO cells, GM-CSF-derived BMDCs; Clinical: Retrospective, 29 advanced solid tumor patients	Peng et al., 2025 (82)
Anti-PD-1	Alistipes finegoldii	A. finegoldii enhances anti-PD-1 efficacy via CXCL16-CXCR6 axis; lipoprotein LIPOAF binds TLR2 to activate NF-kappaB in CCR7+ cDCs, recruiting CXCR6+ CD8+ T cells into TME.	*In vivo*: C57BL/6 mice with MC38 colorectal cancer, B16-F10 melanoma, and additional syngeneic solid tumor models; *In vitro*: BMDCs, splenocytes, tumor cell lines	Wu et al., 2025 (86)

BMDCs, bone marrow-derived dendritic cells; cDCs, conventional dendritic cells; cGAMP, cyclic GMP-AMP; cGAS, cyclic GMP-AMP synthase; CTLA-4, cytotoxic T-lymphocyte-associated antigen-4; DCR, disease control rate; FMT, fecal microbiota transplantation; FLT3L, FMS-like tyrosine kinase 3 ligand; GM-CSF, granulocyte-macrophage colony-stimulating factor; ICI, immune checkpoint inhibitor; IFN, interferon; LGG, *Lactobacillus rhamnosus* GG; mOS, median overall survival; mPFS, median progression-free survival; mRCC, metastatic renal cell carcinoma; MSS, microsatellite-stable; NF-κB, nuclear factor kappa B; NR, not reached; NSCLC, non-small cell lung cancer; ORR, objective response rate; PD-1, programmed cell death protein-1; PD-L1, programmed death-ligand 1; STING, stimulator of interferon genes; TME, tumor microenvironment; TLR2, Toll-like receptor 2.

## 5 Microbial metabolites as mediators of ICI response and toxicity

The human gastrointestinal tract harbors a complex ecosystem composed of bacteria, archaea, viruses, and fungi, collectively known as the gut microbiota (45). Within this biological milieu, microbial metabolites serve as indispensable conduits for host–microbe crosstalk, substantially influencing general physiological homeostasis and disease pathogenesis. Key metabolic classes, including bile acids, SCFAs, branched-chain amino acids, tryptophan, and indole derivatives, have been implicated in a wide range of human pathologies (45–47). As the clinical application of ICIs continues to expand, the interplay between these microbial byproducts and therapeutic outcomes is increasingly being scrutinized.

### 5.1 Tryptophan metabolites: dual modulators of efficacy and toxicity

Tryptophan serves as a primary substrate for microbial metabolism. When tryptophan escapes host absorption in the small intestine, it is converted by the colonic microbiota into various indole-based metabolites, notably 3-IAld and IPA (48). These two metabolic pathways form a systemic synergistic framework that not only bolsters the antitumor activity of ICIs but also selectively ameliorates treatment-induced adverse events, such as colitis and myocarditis.

Renga et al. utilized animal models and indicated that 3-IAld possesses dual functionality: it simultaneously alleviates ICI-induced colitis and enhances antitumor efficacy. This is achieved through the activation of the AhR/IL-22 pathway, which reinforces the epithelial barrier and enriches butyrate-producing bacteria (e.g., *Roseburia*), leading to elevated fecal butyrate levels—a protective effect confirmed by FMT experiments (49). In murine models, Bender et al. demonstrated that *Lactobacillus reuteri*-derived 3-IAld suppresses melanoma growth, extends survival, and potentiates ICI effects (50).

Consistent with these findings, IPA has emerged as a cardioprotective agent. By binding to the aryl hydrocarbon receptor (AhR) and facilitating its nuclear translocation, IPA promotes PI3K expression (51). In recent experimental studies, leflunomide was shown to induce microbiota remodeling to elevate IPA levels. IPA binding to AhR promotes the transcription of the PI3K catalytic subunit-α, subsequently triggering AKT/GSK3β signaling and mitigating PD-1 inhibitor-induced cardiomyocyte apoptosis (52). The discovery of this microbiota–IPA–anti-apoptotic axis is particularly compelling. Both tryptophan and IPA converge on AhR, suggesting that this receptor functions as a master rheostat for microbial metabolites in the regulation of immune and tissue homeostasis. However, whether baseline levels of 3-IAld and IPA can predict the risk of ICI-related colitis or cardiotoxicity remains unknown and requires further clinical validation.

In addition to 3-IAld and IPA, indole-3-lactic acid (ILA), generated by *Bifidobacterium* and *Lactobacillus* species, has emerged as a mediator of intestinal barrier homeostasis. Yu et al. demonstrated that bacterial ILA suppresses CCL2/7 expression in epithelial cells, thereby inhibiting proinflammatory macrophage infiltration and maintaining mucosal quiescence (53). Cui et al. reported that *B. bifidum*-derived ILA ameliorates DSS-induced colitis through AhR/Nrf2 pathway activation, upregulation of tight junction proteins, and suppression of NLRP3 inflammasome activation (54). Ehrlich et al. observed that ILA correlates with *Bifidobacterium*-dominated microbiota and exerts anti-inflammatory effects in intestinal epithelial cells (55). In the context of ICIs, these barrier-protective properties suggest that ILA may counterbalance checkpoint inhibitor-induced intestinal toxicity. Whether baseline fecal ILA levels predict the incidence or severity of irAEs, including colitis, requires direct investigation in immunotherapy cohorts.

### 5.2 Short-chain fatty acids: systemic regulators of immunity and organ protection

Short-chain fatty acids (SCFAs)—primarily butyrate, propionate, and acetate—represent the most abundant class of bacterial fermentation products derived from dietary fiber and complex carbohydrates. Beyond their established role as energy substrates for colonocytes, SCFAs function as pleiotropic immunometabolic signals that bridge gut microbial ecology with systemic antitumor immunity and organ protection. Through distinct receptor-dependent and epigenetic mechanisms, these metabolites modulate macrophage polarization, enhance cytotoxic T-cell function, and reinforce epithelial barrier integrity. Butyrate, in particular, exerts cardioprotective effects against ICI-associated cardiotoxicity via PPARα-mediated NF-κB suppression while simultaneously upregulating antigen presentation machinery in tumor cells through histone deacetylase inhibition. Propionate and acetate, acting primarily through GPR43 (FFAR2), foster regulatory T-cell homeostasis and memory CD8+ T-cell differentiation, thereby calibrating the balance between immune activation and tolerance. The following subsections dissect the molecular pathways through which individual SCFAs potentiate ICI efficacy and mitigate organ-specific adverse events.

#### 5.2.1 Butyrate

Butyrate, a prominent SCFA, has been identified as a critical modulator of PD-1/PD-L1 inhibitor-related cardiotoxicity via the PPARα-CYP4X1 axis (56). As a natural agonist of peroxisome proliferator-activated receptor alpha (PPARα) (57), butyrate suppresses the binding of the transcription factor p65 to NF-κB response elements. This biochemical blockade inhibits the polarization of pro-inflammatory M1 macrophages, thereby reducing TNF-α and IL-1β secretion and cardiomyocyte apoptosis (56).

In addition to its protective role, butyrate acts as an endogenous histone deacetylase (HDAC) inhibitor. Through epigenetic reprogramming, butyrate upregulates the expression of major histocompatibility complex molecules on the surface of tumor cells, enhancing antigen presentation and rendering malignant cells more “visible” to the immune system (58). Simultaneously, butyrate directly influences T-cell dynamics by promoting differentiation into effector T cells and bolstering the infiltration, survival, and cytolytic functions of cytotoxic T lymphocytes within the TME (11). By bridging the gaps between gut metabolism, epigenetic regulation, and tumor immunology, butyrate functions as a systemic regulator that optimizes the safety and efficacy of cancer immunotherapy.

#### 5.2.2 Propionate and acetate

While butyrate has been extensively characterized, propionate and acetate—the remaining principal SCFAs—exert distinct immunomodulatory effects that warrant separate consideration. Bachem et al. demonstrated that propionate and butyrate promote the acquisition of memory potential in antigen-activated CD8+ T cells, thereby augmenting cytotoxic function and long-term survival (59). Smith et al. established that acetate and propionate signal through GPR43 (FFAR2) on colonic immune cells to facilitate extrathymic Treg differentiation, thereby restraining NF-κB-driven inflammation and preserving epithelial barrier integrity (60). Whether the relative abundance of propionate versus acetate confers differential protection against specific irAEs, and whether this ratio can be leveraged as a predictive biomarker, remains to be tested in prospective cohorts.

### 5.3 Potentiating metabolites: TMAO and inosine

While tryptophan catabolites and SCFAs modulate immunity through largely immunoregulatory or barrier-protective mechanisms, the gut microbiota also produces metabolites that directly amplify antitumor cytotoxicity. TMAO and inosine represent two functionally distinct classes—choline-derived and purine-derived, respectively—that converge on enhancing ICI efficacy through non-overlapping signaling nodes. TMAO triggers immunogenic tumor cell death via PERK-GSDME pyroptosis, whereas inosine acts as a conditional immunopotentiator through the adenosine A2A receptor to promote Th1 differentiation during PD-1 blockade. The sections below detail how these metabolites remodel the tumor microenvironment and potentiate checkpoint inhibitor responses.

#### 5.3.1 Trimethylamine N-oxide

In contrast to the supportive roles of indoles and butyrate, TMAO has a more aggressive antitumor mechanism. Rather than simply aiding T-cell function, TMAO acts directly on tumor cells to induce pyroptosis—a highly inflammatory form of programmed cell death. In a large multi-omic analysis of 360 patients with triple-negative breast cancer receiving anti-PD-1 therapy, Wang et al. found that high plasma TMAO levels were associated with superior clinical responses (higher partial response rates) and prolonged PFS (61).

Mechanistically, TMAO triggers endoplasmic reticulum stress by activating protein kinase RNA-like endoplasmic reticulum kinase (PERK), which initiates a signaling cascade that culminates in Gasdermin E (GSDME)-mediated pyroptosis. Specifically, PERK activation facilitates caspase-3 cleavage by GSDME, releasing its N-terminal domain and forming pores in the plasma membrane. This results in cellular swelling, membrane rupture, and the massive release of inflammatory cytokines, such as IL-1β and IL-18 (62). The resulting inflammatory milieu recruits and activates CD^8+^ T cells, while the released tumor antigens and damage-associated molecular patterns stimulate DC maturation and cross-presentation (63). This process stimulates the transition from an immunologically barren to active TME, suggesting that TMAO may offer a strategy to overcome primary resistance in patients unresponsive to ICIs.

#### 5.3.2 Inosine

Beyond SCFAs and indole derivatives, the purine metabolite inosine represents a distinct class of microbiota-derived signals that modulate the TME. Mager et al. revealed that inosine produced by commensal *Bifidobacterium* species engages the adenosine A2A receptor (A2AR) on T cells to promote Th1 differentiation and amplify antitumor immunity during PD-1 blockade (64). This effect requires concurrent TCR signaling, indicating that inosine acts as a conditional immunopotentiator rather than a standalone mitogen. The broader role of adenosinergic signaling in immune regulation has been reviewed by Cekic and Linden, who described how purinergic cues reshape lymphocyte fate at the host–microbe interface (65). In preclinical models of low endogenous *Bifidobacterium* abundance, inosine administration enhanced CD8+ T-cell effector function and improved therapeutic outcomes. These findings identify inosine as a candidate postbiotic for bridging gut microbial ecology with systemic antitumor immunity.

### 5.4 Protective metabolites: secondary bile acids and polyamines

In contrast to metabolites that drive tumor-targeting inflammation, the microbiome generates secondary bile acids and polyamines that primarily constrain collateral immune-mediated tissue damage. These metabolites operate not by enhancing cytotoxicity, but by fortifying epithelial barriers, suppressing NF-κB-driven inflammation, and promoting reparative macrophage polarization—effects that are particularly relevant to the prevention of ICI-induced colitis and systemic irAEs. The following subsections delineate how bile acid biotransformation products and microbial polyamines maintain mucosal and immune homeostasis under checkpoint blockade.

#### 5.4.1 Secondary bile acids

The gut microbiota-mediated conversion of primary bile acids into secondary species, including deoxycholic acid (DCA), generates immunoregulatory signals that intersect with ICI biology. Zhao et al. showed that DCA activates the TGR5 receptor, triggering a cAMP-PKA-dependent cascade that suppresses NF-κβ activation and NLRP3 inflammasome maturation (66). Campbell et al. identified that 3-oxo-LCA and isoallo-LCA regulate peripheral Treg differentiation through the FXR receptor (67). The anti-inflammatory architecture of this system was originally delineated by Guo et al., who demonstrated that TGR5 ligation prevents NLRP3 assembly via PKA-mediated ubiquitination (68). These data suggest that secondary bile acids may dampen systemic inflammation to protect against irAEs while indirectly supporting antitumor immunity through Treg-mediated tolerance. However, the concentration thresholds required for distal organ effects in humans remain undefined.

#### 5.4.2 Polyamines

**Figure 1:**
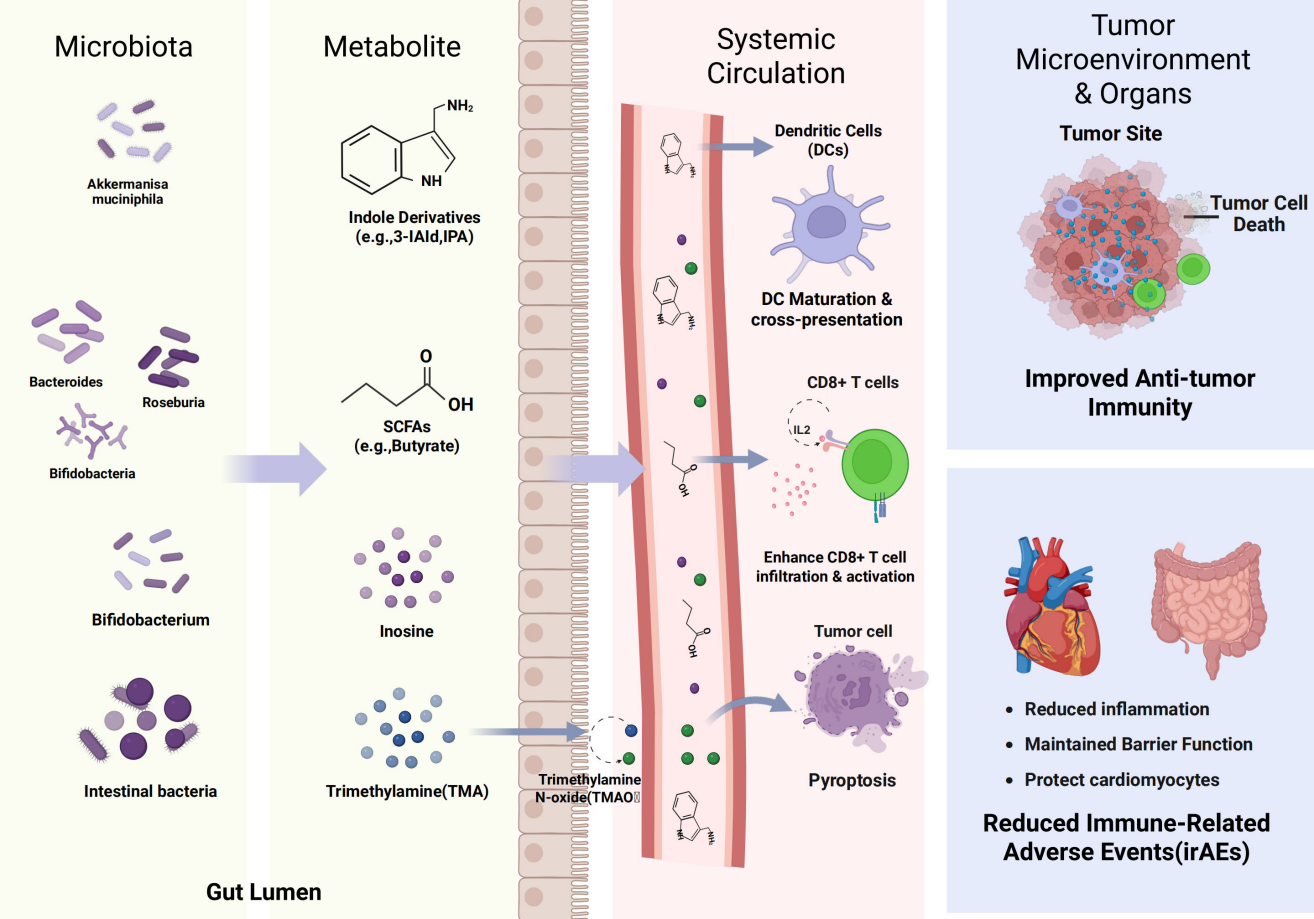
The systemic “Gut-Metabolite-Immune” axis: A framework for balancing ICI efficacy and organ toxicity. The schematic integrates the complex interplay between gut microbiota, their metabolic outputs, and host organ systems. The left panel identifies key bacterial genera and their primary metabolites, including short-chain fatty acids (SCFAs), indole derivatives (e.g., 3-IAld, IPA), and trimethylamine N-oxide (TMAO). The middle panel depicts the translocation of these metabolites via systemic circulation to modulate immune functions, such as AHR-mediated T cell activation. The right panel demonstrates the dual outcomes of this axis: the enhancement of anti-tumor potency at the tumor site and the simultaneous protection of distal organs (e.g., heart and colon) by maintaining barrier integrity and reducing inflammation, thereby mitigating irAEs. SCFAs, short-chain fatty acids; 3-IAld, indole-3-aldehyde; IPA, indole-3-propionic acid; TMAO, trimethylamine N-oxide; AHR, aryl hydrocarbon receptor; irAEs, immunerelated adverse events. This figure was created using BioRender (biorender.com).

Polyamines, including putrescine, spermidine, and spermine, are synthesized by commensal species such as *E. coli*, *Bacteroides*, and *Lactobacillus*, and have long been implicated in epithelial turnover. In the context of ICI toxicity, metagenomic analyses have revealed that baseline enrichment of polyamine transport and biosynthesis pathways in the gut microbiome is associated with resistance to ipilimumab-induced colitis in patients with metastatic melanoma (69). This protective association likely reflects the capacity of polyamines to sustain mucosal integrity under inflammatory stress. Microbial polyamines may reinforce epithelial tight junctions and promote reparative M2 macrophage polarization; however, direct interventional evidence in human ICI cohorts is currently lacking. Direct interventional evidence in human ICI cohorts is currently lacking, and future studies should determine whether polyamine supplementation or engineered polyamine-producing probiotics can attenuate checkpoint inhibitor-induced mucosal injury. While the preceding sections delineate molecular mechanisms by which specific metabolites modulate ICI efficacy and protect against organ-specific toxicities, clinical cohort studies have revealed that these protective associations are highly context-dependent at the community level. The following section examines how baseline microbiome composition predicts irAE risk across diverse cancer types and ICI regimens, and underscores the clinical imperative to decouple efficacy from toxicity. The overall conceptual framework of the systemic ‘Gut–Metabolite–Immune’ axis and its role in balancing ICI efficacy and organ-specific toxicities is illustrated in **Figure 1**.

## 6 Clinical heterogeneity of microbiome-irAE associations

Despite the unprecedented clinical success of ICIs, their utility is frequently hampered by various irAEs, which can preclude many patients from realizing the full treatment benefits. Defined as toxicities with a presumptive immunological etiology, irAEs arise when the reactivation of effector T cells—intended to eliminate malignant cells—results in the collateral overactivation of the immune system, leading to autoimmune-like damage to healthy tissues, commonly affecting the endocrine glands, gastrointestinal tract, lungs, skin, musculoskeletal system, and myocardium. While most irAEs are acute and typically managed with glucocorticoids, this clinical necessity introduces a secondary dilemma: the potential for systemic immunosuppression to attenuate the antitumor efficacy of ICIs.

Longitudinal cohort data from patients with locally advanced or metastatic melanoma have highlighted an efficacy–toxicity trade-off. For instance, the combination of nivolumab and ipilimumab demonstrated a remarkable 10-year OS rate of 43%, significantly outperforming nivolumab (37%) and ipilimumab monotherapies (19%). However, this survival advantage was coupled with a staggering 59% incidence of grade 3–4 treatment-related AEs, exceeding the incidence rates of 23% and 28% in the respective monotherapy arms (70). These findings, which align with previous 5-year survival and toxicity trends (71), underscore a fundamental challenge in the field: the association between therapeutic potency and systemic toxicity. Combination therapies expand survival boundaries at the cost of severe irAEs in more than half of the patient population. Consequently, identifying novel strategies to decouple efficacy from toxicity is imperative for future ICI development.

In this context, the gut microbiota has emerged as a promising candidate for enhancing ICI efficacy while attenuating irAE severity. A growing body of preclinical and clinical evidence suggests that microbial composition can serve as both a predictor and modulator of toxicity. A higher abundance of the phylum *Firmicutes* has been linked to increased toxicity risk, whereas the phylum *Bacteroidetes* is often associated with a lower risk (14, 34). This paradigm has been supported by a prospective cohort study (69) that employed 16S rRNA sequencing in patients with melanoma treated with ipilimumab (anti-CTLA-4). Accordingly, patients with a high baseline abundance of *Bacteroidetes* were significantly less likely to develop immune-mediated colitis than those with lower abundance.

However, this protective relationship is reversed in hepatobiliary malignancies. Mao et al. (72) analyzed 65 patients with advanced hepatocellular carcinoma or cholangiocarcinoma receiving anti-PD-1 monotherapy, of whom 57 developed mild colitis and 8 experienced severe colitis. Metagenomic profiling revealed that the patients with severe colitis possessed a significantly higher proportion of *Bacteroidetes*, whereas those with mild colitis had an abundance of *Firmicutes*. This striking contradiction suggests that in the specific landscape of hepatobiliary tumors, *Firmicutes* may play a dominant role in suppressing intestinal irAEs.

The findings of Mao et al. serve as a critical caution: a “beneficial” microbe in one patient population may be “detrimental” in another. This inconsistency underscores that microbial biomarkers for irAEs must be rigorously validated in organ- and tumor-specific contexts. This further imposes a higher standard for future research, suggesting that a “universal” microbial signature may not exist across different cancers and drug classes. Much like the microbial influence on ICI efficacy, the impact on irAEs is not governed by a universal biological law but is instead a highly context-dependent phenomenon, dictated by a triangulation of tumor type (e.g., melanoma vs. hepatobiliary cancer), specific target pathway (anti-CTLA-4 vs. anti-PD-1), and organ-specific vulnerabilities. Notably, current microbiome-based interventions have primarily focused on enhancing objective response rates; rigorous monitoring of irAE profiles in future probiotic and FMT trials will be essential to determine whether these strategies can indeed decouple antitumor efficacy from autoimmune toxicity. Postbiotic and probiotic-derived bioactive molecules, including siderophores and cyclic dinucleotides, are mechanistically summarized alongside classical metabolites in **Table 2**.

**Table 2:** Bioactive gut microbiota-derived molecules in the bifunctional modulation of ICI antitumor efficacy and toxicity.

Metabolite	Microbial source	Primary action	Mechanism of antitumor efficacy	Mechanism of toxicity modulation	References
3-IAld	L. reuteri, Gut microbiota	Dual	AhR/IL-22 axis enhances antitumor immunity	Alleviates ICI-colitis; reinforces epithelial barrier; restores tight junctions	Renga et al., 2022 (49); Bender et al., 2023 (50)
Butyrate	Roseburia, F. prausnitzii, Prevotellaceae	Dual	HDAC inhibition upregulates MHC/antigen presentation; promotes effector T-cell differentiation and CD8+ T-cell infiltration via ID2-dependent IL-12 signaling	PPARα-CYP4X1 axis inhibits NF-κB; protects cardiomyocytes; promotes Treg differentiation; reduces TNF-α and IL-1β secretion	Chen et al., 2022 (56); Kespohl et al., 2017 (58)
Propionate/Acetate	Bifidobacterium, Faecalibacterium	Dual	Enhances CD8+ T-cell cytotoxicity and memory potential; promotes antigen-activated CD8+ T-cell differentiation	GPR43/FFAR2 signaling facilitates extrathymic Treg differentiation; restrains NF-κB-driven inflammation; maintains barrier integrity	Bachem et al., 2019 (59); Smith et al., 2013 (60)
TMAO	Gut microbiota	Efficacy	PERK activation triggers GSDME-mediated pyroptosis; recruits CD8+ T cells; converts immunologically cold to hot TME	—	Wang et al., 2022 (61)
c-di-AMP	A. muciniphila	Efficacy	Directly binds and activates host cytosolic STING receptors; triggers robust type I interferon response	—	Lam et al., 2021 (84)
Siderophores	Lactobacillus	Efficacy	c-Jun N-terminal kinase signaling cascade triggers iron-chelation-mediated tumor cell apoptosis	—	Oh et al., 2019 (57)
Inosine	Bifidobacterium	Efficacy	A2AR signaling promotes Th1 differentiation; amplifies antitumor immunity during PD-1 blockade; enhances CD8+ T-cell effector function	—	Mager et al., 2020 (64)
IPA	Gut microbiota	Toxicity	—	AhR-PI3K-AKT-GSK3β axis attenuates PD-1 inhibitor-induced cardiomyocyte apoptosis; promotes PI3K transcription and nuclear translocation of AhR	Huang et al., 2025 (52)
ILA	Bifidobacterium, Lactobacillus	Toxicity	—	AhR/Nrf2 pathway upregulates tight junction proteins; suppresses NLRP3 inflammasome activation; inhibits CCL2/7-driven proinflammatory macrophage infiltration	Yu et al., 2023 (53); Cui et al., 2023 (54); Ehrlich et al., 2020 (55)
Secondary bile acids	Firmicutes, Bacteroides	Toxicity	—	DCA activates TGR5-cAMP-PKA cascade suppressing NF-κB/NLRP3; 3-oxo-LCA and isoallo-LCA regulate peripheral Treg differentiation via FXR receptor	Zhao et al., 2023 (66); Campbell et al., 2020 (67); Guo et al., 2016 (68)
Polyamines	E. coli, Bacteroides, Lactobacillus	Toxicity	—	Baseline enrichment of polyamine transport and biosynthesis pathways is associated with resistance to ipilimumab-induced colitis	Dubin et al., 2016 (69)

3-IAld, indole-3-aldehyde; IPA, indole-3-propionic acid; ILA, indole-3-lactic acid; TMAO, trimethylamine N-oxide; c-di-AMP, cyclic di-adenosine monophosphate; A2AR, adenosine A2A receptor; AhR, aryl hydrocarbon receptor; HDAC, histone deacetylase; PPARα, peroxisome proliferator-activated receptor alpha; NF-κB, nuclear factor kappa B; STING, stimulator of interferon genes; PERK, protein kinase RNA-like endoplasmic reticulum kinase; GSDME, gasdermin E; TME, tumor microenvironment; DCA, deoxycholic acid; TGR5, G protein-coupled bile acid receptor 1; FXR, farnesoid X receptor; IL, interleukin; TNF-α, tumor necrosis factor-alpha; Treg, regulatory T cell; ICI, immune checkpoint inhibitor.

## 7 Microbiome-based interventions

The conceptual framework of probiotics—defined by the Food and Agriculture Organization/World Health Organization (FAO/WHO) in 2001 as “live microorganisms which, when administered in adequate amounts, confer a health benefit on the host” (73)—originated from early observations linking lactic acid bacteria in yogurt to human longevity (74). To exert a meaningful physiological effect within the human gut, these microbes must typically reach a viable concentration of at least 10^6^ CFU/mL in the small intestine and 10^8^ CFU/g in the colon (73). Achieving such thresholds, however, is a formidable challenge because the harsh gastrointestinal milieu, characterized by low gastric pH, enzymatic degradation, and antimicrobial activity of bile salts, serves as a significant barrier to effective delivery. Traditional administration methods are frequently ineffective owing to low microbial survival rates and a lack of site-specific targeting, thereby hindering the broad clinical adoption of probiotic therapies.

Early clinical evidence for the benefits of probiotics emerged in 2016 when Yang et al. demonstrated that perioperative supplementation with a multispecies probiotic (including *Bifidobacterium*, *Lactobacillus*, and *Enterococcus*) significantly accelerated intestinal recovery in patients following colorectal cancer resection (75). Subsequently, Huang et al. found that a probiotic combination incorporating *L. acidophilus* and *B. infantum* mitigated chemotherapy-induced gastrointestinal complications, specifically diarrhea, and promoted the production of SCFAs, suggesting fundamental remodeling of the gut microbiota (76).

The recent evolution of encapsulation technologies has begun to systematically dismantle the triple barrier of gastric acid inactivation, bile salt toxicity, and colonization failure. Pan et al. developed a nano-armor using a single-cell metal–organic network, which achieved a 78% survival rate for *Lactobacillus rhamnosus* GG (LGG) in pH 2.5 gastric juice—a stark contrast to the 1% survival of uncoated bacteria—and enhanced colonic colonization in mice by over three orders of magnitude (77). Similarly, Wu et al. utilized bovine exosome-chitosan hybrid membranes to increase the mucus penetration rate of *B. longum* by 3.6-fold (78), whereas Xie et al. reported that pectin–calcium alginate dual-network microspheres could extend the shelf life of *L. plantarum* and maintain a high output even after passing through simulated gastrointestinal models (79). These engineering breakthroughs have shifted the paradigm of probiotic delivery from gram-scale loss to logarithmic redundancy. However, this technological leap introduces a new challenge: the engineering of delivery vehicles has outpaced the functional validation of the probiotic cargo itself. Future clinical success will depend on matching how a microbe is delivered with precise understanding of which specific strains are effective. The distinct immunomodulatory pathways of live probiotics, postbiotics, and microbial metabolites are comparatively outlined in **Table 2**.

### 7.1 Probiotics and defined strains

Based on their molecular targets, immunomodulatory probiotics can be broadly categorized into three mechanistic classes: (i) cytosolic DNA-sensing and STING activators; (ii) peptidoglycan- and chemokine-axis modulators; and (iii) adaptive immune-trafficking regulators. Representative examples of each class are discussed below.

#### 7.1.1 LGG amplifies anti-PD-1 efficacy via the cGAS-cGAMP-STING axis

LGG is among the most extensively studied probiotics in clinical use. Oral administration of live LGG was shown to significantly enhance the response to anti-PD-1 therapy in murine models of melanoma and colon cancer (80). Mechanistically, LGG triggers the production of IFN-β in DCs by activating the cGAMP-STING axis. STING serves as a critical sensing hub for cytosolic DNA, which, in the context of malignancy, often arises from genomic instability or treatment-induced damage. Activation of the cGAS-cGAMP-STING pathway triggers type I interferons and a pro-inflammatory cascade that remodels the TME, increasing the infiltration and activity of CD^8+^ T cells, NK cells, and DCs (81).

Once ingested, LGG is rapidly captured by intestinal DCs, and its genomic DNA triggers the intracellular cGAS-STING-TBK1-IRF7 cascade. This signaling not only maintains the cross-presentation capabilities of DCs in a high-alert state but also activates CD^8+^ T cells via autocrine and paracrine pathways, driving their recruitment into the tumor and subsequent transformation into a cytotoxic phenotype high in IFN-γ and granzyme B. Notably, this passive presentation model—where the host DCs must actively take up LGG to release its internal DNA—suggests that therapeutic efficiency is highly dependent on the phagocytic functions of the host, which have significant interpatient variability. While these actions position live probiotics as natural STING agonists, they also account for potential heterogeneity in clinical outcomes. Furthermore, microbial analysis showed that LGG treatment (alone or in combination with anti-PD-1) shifted the microbiota toward a healthier composition, characterized by an increase in *Bacteroidetes* and a decrease in *Verrucomicrobia (*80).

#### 7.1.2 BF839 synergizes with anti-PD-1 via cGAS-STING activation

Like LGG, the probiotic Bacteroides *fragilis* BF839 operates through a related yet distinct modality of cGAS-STING activation. In C57BL/6 mice bearing B16-F10 melanoma, oral administration of BF839 significantly inhibited tumor growth and synergized with anti-PD-1 antibody to induce tumor regression. Mechanistically, RNA sequencing and STING knockout validation revealed that BF839-mediated tumor suppression is strictly dependent on the cGAS-STING pathway—an effect completely abolished in B16-STING-KO tumors. This pathway activation coincided with markedly increased CD8^+^ T cell infiltration within the TME, pointing to enhanced downstream type I interferon responses (82). Notably, a retrospective clinical evaluation of 29 patients with advanced solid tumors receiving BF839 as long-term adjuvant therapy alongside ICIs and chemotherapy demonstrated improved OS compared to short-term therapy recipients, positioning BF839 as a clinically translatable STING-activating probiotic (82).

#### 7.1.3 *Akkermansia muciniphila* (Akk)-Derived c-di-AMP: Direct STING Activation

**Figure 2:**
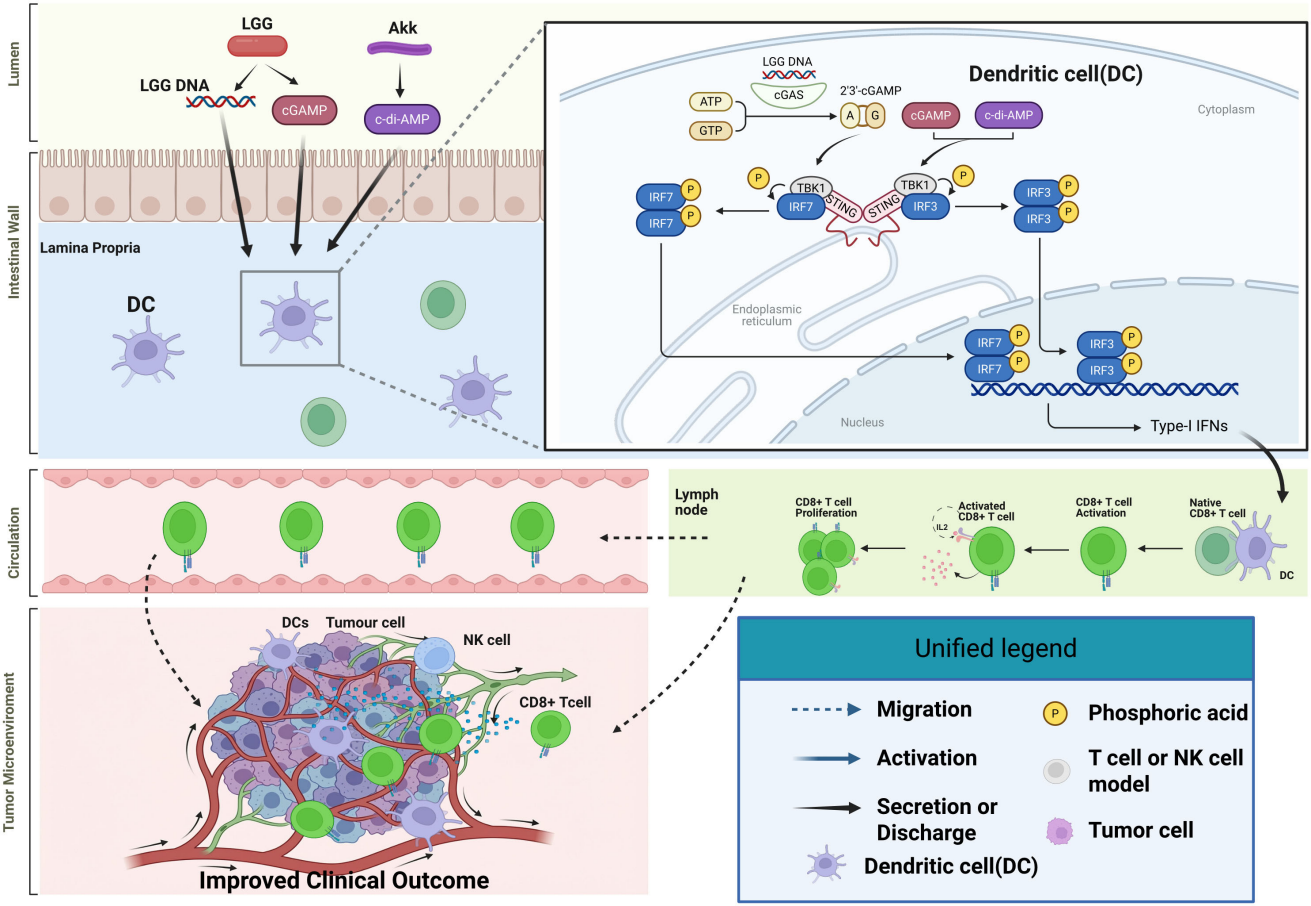
Molecular orchestration of the cGAS-STING axis by microbe-derived signals to enhance systemic anti-tumor immunity. This figure delineates the molecular cascade through which intestinal commensals prime the host immune system. The upper panel details the intracellular sensing of microbial signals (e.g., LGG genomic DNA or Akkermansia-derived c-di-AMP) within intestinal dendritic cells (DCs), leading to the activation of the cGAS-cGAMP-STING pathway and subsequent production of Type I interferons (IFN-b). The lower panel illustrates the systemic amplification of this signal: microbial-induced IFNs drive DC maturation and antigen cross-presentation, facilitating the activation, proliferation, and systemic migration of CD8+ T cells from the lymph nodes to the tumor microenvironment to enhance tumor cell killing. DC, dendritic cell; cGAS, cyclic GMP-AMP synthase; STING, stimulator of interferon genes; cGAMP, cyclic GMP-AMP; c-di-AMP, cyclic di-adenosine monophosphate; IFN, interferon; TCR, Tcell receptor. This figure was created using BioRender (biorender.com).

Compared to the passive actions of LGG on STING signaling, Akk appears to have a more active mechanism. Lam et al. observed superior antitumor effects in germ-free mice monocolonized with Akk. Both *in vitro* cultures and *in vivo* cecal contents from these mice revealed high levels of cyclic di-AMP (c-di-AMP), a bacterium-derived cyclic dinucleotide. Crucially, c-di-AMP can directly bind to and activate host cytosolic STING receptors, bypassing the requirement for complete bacterial phagocytosis. This allows the effector molecules to diffuse and act on a broad range of target cells. However, the stability and bioavailability of c-di-AMP within the complex intestinal environment and whether it reaches the concentration threshold required for STING activation in humans remain unclear (83, 84). The distinct molecular pathways through which different bacterial species, such as LGG and Akk, activate the host cGAS-STING axis to modulate antitumor immunity are schematically illustrated in **Figure 2**. Beyond STING-dependent cytosolic sensing, other commensals exploit distinct pattern-recognition receptors or chemokine axes to potentiate ICI efficacy.

#### 7.1.4 The SagA-NOD2 axis: enhancing anti-PD-L1 via peptidoglycan hydrolysis

Specific strains of *Enterococcus*, such as *E. faecium* Com15, markedly potentiate anti-PD-L1 therapy through the expression of SagA, a conserved NlpC/p60 peptidoglycan hydrolase. SagA cleaves peptidoglycans into smaller immunoactive muropeptides (such as glucosaminyl muramyl dipeptide and MDP), which are subsequently recognized by the nucleotide-binding oligomerization domain-containing protein 2 (NOD2) receptor in host immune cells (85). Activation of the downstream NF-κB and mitogen-activated protein kinase pathways results in enhanced antitumor immunity. The central role of this enzyme was demonstrated by introducing the *sagA* gene into an otherwise ineffective *E. faecalis*, which subsequently acquired the ability to enhance immunotherapy, confirming that SagA is the master regulator of this axis.

#### 7.1.5 *Alistipes finegoldii* enhances anti-PD-1 efficacy through the CXCL16-CXCR6 axis

Recent investigations have expanded the repertoire of immunomodulatory commensals beyond classical STING and NOD2 agonists. *Alistipes finegoldii*, a gram-negative anaerobe enriched in responders across multiple cancer cohorts, was shown to augment anti-PD-1 efficacy in C57BL/6 mice bearing MC38 colorectal cancer and B16-F10 melanoma. Rather than acting through pattern recognition receptors alone, *A. finegoldii* secretes a lipoprotein termed LIPOAF, which binds Toll-like receptor 2 (TLR2) on CCR7+ conventional DCs. This interaction activates NF-κB signaling and upregulates CXCL16 expression, thereby recruiting CXCR6-expressing CD8+ T cells into the TME. This discovery establishes a microbiota-driven chemotactic axis that directly links gut commensals to intratumoral T-cell trafficking (86).

#### 7.1.6 *L. bulgaricus* EPS-R1: modulation via the LPA2-CCR6 axis

In contrast to the innate-focused pathways described above, *Lactobacillus bulgaricus* OLL1073R-1 primarily acts on the adaptive immune system. In tumor models expressing CCL20, exopolysaccharide (EPS-R1) produced by this strain synergizes with anti-CTLA-4 or anti-PD-1 antibodies to suppress tumor growth and increase the infiltration of CCR^6+^ CD^8+^ T cells (87).

Mechanistically, the glycerol-3-phosphate structure of EPS-R1 binds to lysophosphatidic acid receptor 2 (LPA2) in CD^8+^ T cells. This binding activates the LPA2 receptor, which prevents the downregulation of CCR6 typically caused by inhibitory receptors, such as LPA5. Consequently, these CCR^6+^ CD^8+^ T cells migrate more efficiently toward the tumor tissue via the CCL20-CCR6 axis. By bypassing classical pattern recognition receptors (such as STING or NOD2) and acting as atypical ligands for LPA2, EPS-R1 demonstrates that probiotics can functionally regulate the immune system.

#### 7.1.7 Clinical translation: from murine mechanisms to randomized trials

Translating preclinical probiotic mechanisms into randomized clinical settings, Ebrahimi et al. reported a Phase I trial in patients with metastatic renal cell carcinoma receiving cabozantinib plus nivolumab, who were randomized to receive a multi-strain live bacterial consortium or placebo. This study provided early clinical evidence that live bacterial supplementation can modulate the gut microbiota and potentially enhance ICI efficacy within a targeted therapy backbone, bridging the gap between murine models and human randomized controlled trials (88). Collectively, the above evidence demonstrates that different probiotics may target distinct stages of the antitumor response—such as priming, expansion, or recruitment—providing a theoretical foundation for future probiotic cocktail therapies.

### 7.2 Therapeutic potential of FMT in enhancing ICI efficacy

While defined probiotics and their purified effectors offer strain-specific precision, their efficacy is constrained by colonization resistance, interpatient variability in host phagocytic uptake, and the finite immunomodulatory spectrum of single-species administration. Fecal microbiota transplantation (FMT), the process of transferring fecal flora from a healthy donor to the patient’s gastrointestinal tract to restore a balanced microbial ecosystem, represents a more radical approach to ecosystem restructuring. Originally pioneered for the treatment of refractory *Clostridioides difficile* infection (CDI) (89), FMT has attracted growing interest in oncology following the discovery of the gut–immune axis.

The potential of FMT to reverse therapeutic resistance was further underscored in a small but pivotal cohort study involving 10 patients with metastatic melanoma who were previously unresponsive to anti-PD-1 therapy (90). FMT reinvigorated the TME, as evidenced by the enhanced infiltration of APCs and CD^8+^ T cells, resulting in an objective response rate of 30%. Although these initial findings are encouraging, the small sample size necessitates larger and more robust studies to validate the impact of donor variability on treatment efficacy and to define the optimal microbial signatures required for success.

Complementing this work, a single-arm trial involving 15 patients with primary resistant melanoma who underwent FMT sourced from long-term responders to PD-1 therapy in combination with pembrolizumab administration was conducted (91). This combined intervention achieved disease control in 40% of the patients, with median PFS and OS of 14 months. Mechanistically, researchers have observed a systemic shift toward highly activated CD^8+^ T-cell phenotypes (CD56^+^, CD57^+^, TIGIT^+^, and GrB^+^) and the upregulation of granzyme B in mucosa-associated invariant T cells. Intratumorally, there was a marked reduction in IL-8^+^/SPP1^+^ myeloid-suppressor cells and FoxP3^+^ Tregs, along with an increase in CD^8+^ T cells expressing HLA-DR and GZMK. By demonstrating that microbial engraftment coupled with systemic immunometabolic reprogramming can reverse PD-1 resistance, this study provided the first definitive evidence that FMT can reshape the intestinal ecosystem and restore therapeutic efficacy.

Building upon foundational Phase I evidence, the field has advanced to randomized Phase II trials. The multicenter Phase II FMT-LUMINate trial enrolled 40 patients—20 treatment-naive patients with NSCLC (PD-L1 ≥ 50%) and 20 with melanoma—who underwent FMT from healthy donors followed by anti-PD-1 monotherapy or dual anti-PD-1 plus anti-CTLA-4. The objective response rate reached 80% in the NSCLC cohort and 75% in the melanoma cohort. Shotgun metagenomic sequencing revealed that clinical response was associated with the loss of deleterious baseline species, including *Enterocloster*, *Streptococcus*, and *Clostridium* spp., rather than simple donor-strain engraftment. This finding has been reproduced in three published FMT oncology trials and validated in murine models, where reintroduction of lost bacterial species abrogated the antitumor effect of ICIs. These data suggest that FMT efficacy may depend equally on “weeding out” harmful taxa as on engrafting beneficial ones (92).

Parallel efforts have extended FMT applications beyond melanoma to gastrointestinal malignancies. Zhang et al. conducted a Phase I trial (NCT04130763) in 10 patients with ICI-refractory microsatellite-stable gastric or colorectal cancer, in whom FMT combined with nivolumab therapy was conducted. The regimen achieved an objective response rate of 20% and a disease control rate of 40% in a population traditionally resistant to immunotherapy. Donor-derived immunogenic microbes, including *Bacteroides stercoris* and *Prevotella* spp., were identified as potential mediators of resensitization; the microbial signature was independently validated in a separate cohort of 63 patients with gastric cancer, where responder-enriched microbes outperformed non-responder taxa in predicting anti-PD-1 sensitivity (93).

When analyzed in tandem, the trials by Baruch et al. and Davar et al. highlight fecal donor selection as a critical variable in treatment efficacy. While Baruch et al. utilized healthy donors and observed significant inter-individual variability in treatment response, Davar et al. achieved a higher objective response rate by strategically selecting long-term responders as donors. This comparison suggests that the success of FMT may not only depend on restoring a healthy or normal microbiome but rather on transplanting a functional consortium that has been vetted by prior ICI exposure. Thus, an ideal donor must be healthy and possess specific microbial features capable of driving antitumor immunity.

## 8 Synergistic regulation of ICI efficacy through diet–microbiome interplay

Beyond administering live microbes or entire ecosystems, modulating the microbiome through diet and its non-volatile fermentation products represents a scalable, non-invasive adjunct to ICI therapy. The following sections discuss postbiotics, dietary fiber, and the emerging paradigm of precision nutrition.

### 8.1 Postbiotics: a non-viable frontier

In a landmark consensus statement, the International Scientific Association for Probiotics and Prebiotics formally codified the term “postbiotic” as a “preparation of inanimate microorganisms and/or their components that confers a health benefit on the host” (94). This definition has led to a surge in research exploring the intersection between postbiotics and oncology. For instance, the Ferrari team identified the significant antitumor potential of phytosphingosine, a key postbiotic component; when combined with anti-PD-1 therapy in murine models, it markedly inhibited tumor progression and extended survival (95). Similarly, MS-20, a soy-fermented broth derived from the symbiotic fermentation of *Lactobacillus* and yeast, suppressed tumor growth when administered alongside PD-1 blockade (96).

The application of postbiotics remains largely exploratory, yet preliminary data are compelling. Cell-free supernatants from *Lactobacillus delbrueckii* increased colorectal cancer cell apoptosis by activating the caspase-3-dependent pathway (50). As detailed in Section 5.2, butyrate and other SCFAs are critical microbial metabolites bridging dietary fiber to host immunity. Here, we focus on non-viable microbial components (postbiotics) that exert immunomodulatory or antineoplastic effects independent of live bacteria. Although the inactivated cells and supernatants of *L. plantarum* A7 and LGG inhibit both normal and malignant colon cell growth, these effects often mirror those of live bacterial interventions, suggesting that viability may not always be a prerequisite for efficacy (56).

Perhaps the most promising approach is probiotic-derived siderophores. These iron-sequestering molecules, particularly those secreted by lactic acid bacteria, exhibit potent anti-neoplastic effects *in vitro* and occasionally outperform conventional chemotherapeutics, such as cisplatin and 5-fluorouracil (5-FU). Mechanistically, siderophores trigger colon cancer cell apoptosis through the c-Jun N-terminal kinase signaling cascade while sparing healthy intestinal cells from growth inhibition (57). Furthermore, siderophores produced by *L. casei* ATCC334 induce gastric cancer cell apoptosis, and their efficacy has been confirmed in 5-FU-resistant PDAC cells (58, 61).

### 8.2 Dietary polysaccharides and the fiber hypothesis

Dietary polysaccharides are characterized by immense structural diversity manifested in their glycosidic bond configurations and varying degrees of polymerization. This complexity allows them to cause predictable and significant shifts in gut microbiota composition (97). Preclinical evidence strongly supports the hypothesis that fiber modulates the microbiome, whereas low-fiber diets or inappropriate probiotic use may dampen antitumor immunity.

In a large-scale study of patients with melanoma receiving ICIs, those who reported high fiber intake (> 20 g/d) exhibited significantly prolonged PFS and superior response rates compared to those with low fiber intake (98). The mechanistic basis of this effect—microbial fermentation of fiber into SCFAs—has been discussed in Section 5.2. Here, we focus on clinical cohort evidence linking dietary fiber intake to ICI outcomes. Although the association between polysaccharides and microbial shifts is well-documented, the mapping of these relationships, specifically to ICI potentiation, remains an emerging field for future investigation.

### 8.3 Precision nutrition: moving beyond universal guidelines

Universal dietary recommendations have yielded inconsistent results across patients with different malignancies, prompting growing interest in precision nutrition tailored to individual gut microbiome profiles. Accumulating evidence indicates that baseline microbial composition substantially determines ICI response (11, 14, 34). Patients whose baseline microbiota is enriched with fiber-fermenting taxa, such as *Ruminococcaceae*, appear to derive greater benefit from high-fiber diets (98), whereas individuals lacking specific metabolic strains (e.g., *Akk* or *B. longum*) are unlikely to respond meaningfully to dietary modification alone (37, 38, 99).

Nevertheless, translating this paradigm into routine clinical practice faces considerable logistical obstacles. Turnaround time represents the most immediate hurdle. Current clinical shotgun metagenomic sequencing—from sample processing and library preparation through sequencing and bioinformatic interpretation—typically requires several days before a clinically actionable report can be issued (100). Although nanopore sequencing can compress the turnaround time for pathogen detection to 7–9 hours (101), its current read depth and degree of standardization remain insufficient to support oncological nutritional decisions based on ecological profiling of microbial communities. When additional delays for sample transport, clinical review, and dietary protocol formulation are factored in, the interval from sample collection to delivery of individualized dietary recommendations approximates 1–2 weeks. This temporal constraint renders precision nutrition better suited as a pre-treatment stratification tool rather than as a modality that can be dynamically adjusted during ongoing therapy.

Another frequently overlooked concern is safety. High-fiber diets are not universally well tolerated. While the study by Spencer et al. (98) demonstrated immunological benefits from high fiber intake, such benefits presuppose adequate fermentative capacity within the gut. In patients whose baseline microbiota is severely depleted of butyrate-producing organisms (e.g., *Faecalibacterium*, *Roseburia*, *E. rectale*), an abrupt increase in dietary fiber intake may overwhelm residual glycolytic fermentation, shifting residual microbial metabolism toward proteolytic pathways. This diversion increases gas production and intestinal distension, manifesting as clinically significant bloating, cramping, and even obstipation (94). In patients with concurrent small intestinal bacterial overgrowth, active immune-related colitis, or severe treatment-associated dysbiosis, such high-fiber loading is not merely ineffective but may exacerbate mucosal inflammation and compromise tolerance to subsequent therapy.

Therefore, the simplistic formula that “high fiber is universally beneficial” should not be applied indiscriminately in clinical practice. A more prudent approach entails initial baseline metagenomic assessment of fiber-fermenting taxa. When such populations are markedly depleted, high-fiber prescriptions should be avoided initially; instead, a stepwise escalation strategy (e.g., increasing fiber by no more than 5 g per week) should be adopted, coupled with targeted probiotic or postbiotic supplementation to precondition the microbial community. Only after partial restoration of fermentative capacity should fiber intake be gradually advanced. By reconciling the practical constraints of testing turnaround with individual safety considerations, precision nutrition may transition from a theoretical construct to a clinically implementable adjunct.

## 9 Discussion

The gut microbiome functions as a systemic regulator of ICI efficacy and toxicity, yet the field remains transitional between observational correlation and mechanistic causality. Preclinical models have established that Bacteroides, Bifidobacterium, and Akkermansia muciniphila potentiate antitumor immunity through cGAS-STING, NOD2, and AhR pathways, but these findings derive largely from murine systems or small cohorts with limited covariate adjustment. Strain-level specificity complicates translation: Bifidobacterium bifidum strains K57 and K18 synergize with PD-1 blockade, whereas other conspecific strains do not. Similarly, Firmicutes and Bacteroidetes associations with irAE risk invert across cancer types—protective in melanoma yet linked to severe colitis in hepatobiliary malignancies—indicating that microbial biomarkers are context-dependent, shaped by tumor type, host genetics, and ICI regimen.

Microbial metabolites modulate immune responses through distinct molecular targets. SCFAs signal via PPARα and HDAC inhibition to enhance antigen presentation and T-cell cytotoxicity; indole derivatives activate AhR to maintain barrier integrity; and TMAO triggers PERK-GSDME-mediated pyroptosis to recruit CD8+ T cells. However, human concentration thresholds for distal organ effects remain undefined, and local concentrations within the tumor microenvironment necessary to drive DC maturation or T-cell recruitment remain uncharacterized. Most evidence describes systemic or intestinal metabolite levels rather than TME-specific dynamics. Resolving these spatial unknowns will require single-cell sequencing, spatial transcriptomics, and patient-derived organoid models to reconstruct the microbiota–metabolite–immune network at cellular resolution.

Clinical translation faces biological and logistical barriers. Encapsulation technologies have achieved substantial improvements in probiotic gastric survival, yet the engineering of delivery vehicles has outpaced functional validation of the microbial cargo. FMT trials demonstrate that responder-derived microbiota outperforms healthy donor material, suggesting that the ideal transplant is not a restored normal flora but a functionally vetted consortium primed by prior ICI exposure; however, the specific microbial features required remain incompletely characterized. Precision nutrition is constrained by metagenomic turnaround times of days to weeks, rendering real-time dietary adjustment impractical during active therapy. In patients depleted of butyrate-producing organisms, abrupt fiber loading may shift residual metabolism toward proteolytic pathways, increasing gas production and mucosal inflammation rather than enhancing immunotherapy response.

Methodological heterogeneity further undermines reproducibility. Contradictory findings stem not only from cohort differences but from discrepancies in sample processing and bioinformatic pipelines. Reporting guidelines such as STORMS remain underutilized, and pre-analytical variables—including storage temperature, transport time, DNA extraction kits, and primer pairs—are frequently omitted. At the wet-laboratory level, routine inclusion of mock communities and positive controls would improve internal quality control and permit cross-laboratory benchmarking of batch effects, while negative controls are necessary to flag reagent contamination in low-biomass fecal studies. The debate over 16S rRNA versus shotgun metagenomics remains unresolved: 16S suffices for taxonomic enrichment and alpha-diversity in large cohorts, whereas shotgun metagenomics—and ideally paired metatranscriptomics—is indispensable for strain-level resolution, functional gene annotation, and metabolomic integration. Establishing clear criteria for matching sequencing strategy to specific research questions would prevent the methodological mismatch that currently confounds cross-study synthesis.

**Figure 3:**
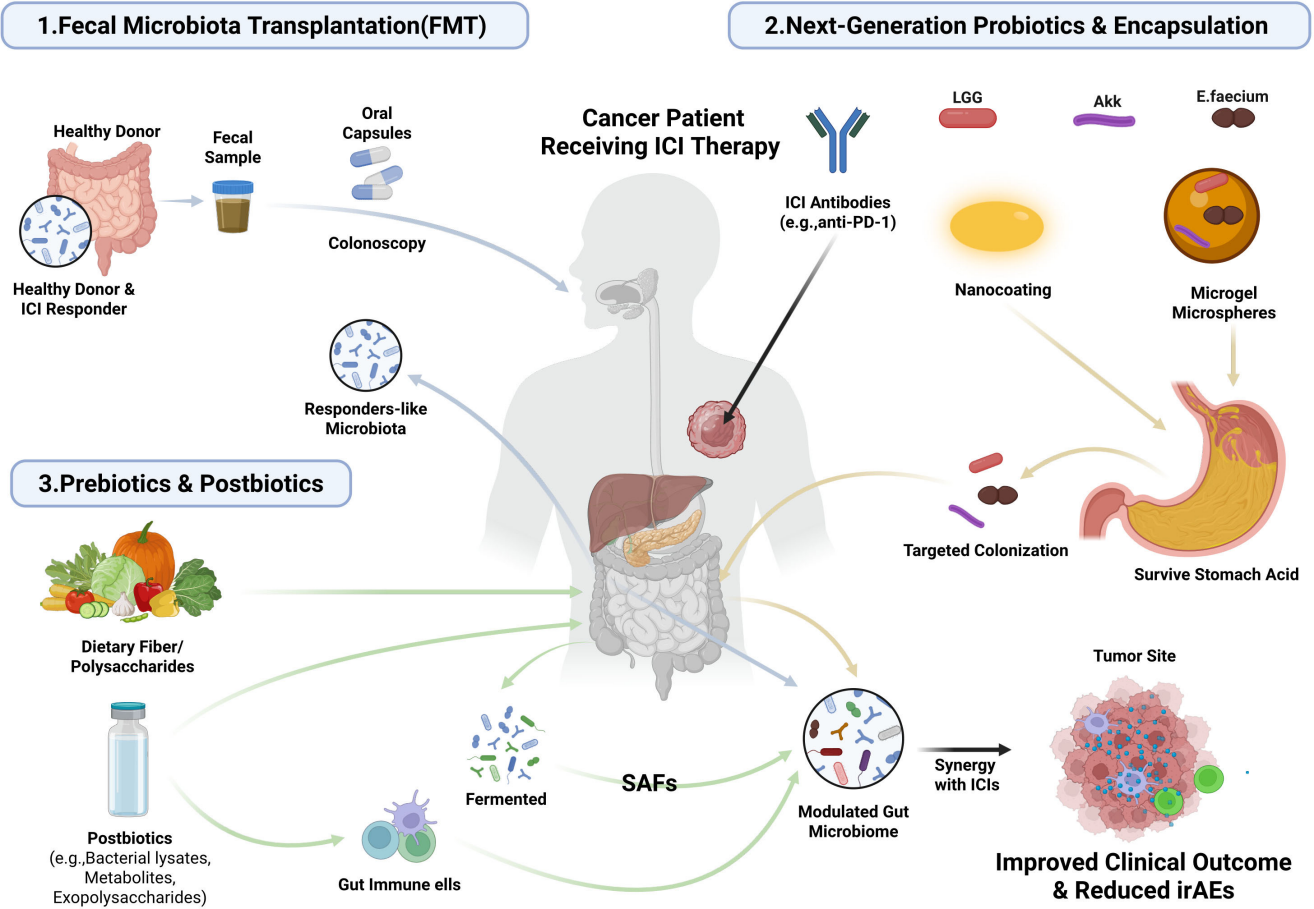
Integrated landscape of precision microbiome-based interventions to optimize immune checkpoint inhibitor (ICI) therapy. This illustration presents a comprehensive framework of microbiome-targeted therapeutic modalities designed to enhance the clinical efficacy of immune checkpoint inhibitors (ICIs) and mitigate immune-related adverse events (irAEs). The therapeutic landscape encompasses a multi-pronged approach: the transplantation of “responder-derived” microbiota through Fecal Microbiota Transplantation (FMT); the targeted delivery of next-generation probiotics (e.g., LGG, Akk, and E. faecium) facilitated by advanced encapsulation technologies like nanocoatings and microgel microspheres to ensure survival against gastric acid; and the adjunctive use of prebiotics (e.g., dietary fibers) and postbiotics (e.g., bacterial lysates and metabolites). These strategies collectively modulate the gut-metabolite-immune axis, fostering a favorable intestinal ecosystem that synergizes with ICI antibodies (e.g., anti-PD-1) to promote anti-tumor immunity at the tumor site while restoring systemic immune homeostasis. ICI, immune checkpoint inhibitor; FMT, fecal microbiota transplantation; LGG, Lactobacillus rhamnosus GG; Akk, Akkermansia muciniphila; irAEs, immune-related adverse events; SCFAs, short-chain fatty acids (and related fermentation products). This figure was created using BioRender (biorender.com).

Analytical frameworks must move beyond single-taxa abundance. Topology-based approaches, such as the ecological scoring system developed by Derosa et al., capture inter-species network relationships and outperform traditional alpha-diversity metrics across multiple cancer types. Future studies should prioritize strain-specific functional validation through advanced culturomics and synthetic consortia, acknowledging that microbial function emerges from community architecture rather than isolated keystone species. Ultimately, the central goal is to develop precise microbial interventions—engineered probiotics, defined metabolite cocktails, or responder-derived FMT—through standardized, multicenter longitudinal studies integrating high-resolution multi-omics with rigorous clinical validation to maximize therapeutic gains while decoupling efficacy from systemic toxicity. The future roadmap for such precision microbial interventions is envisioned in **Figure 3**.

## 10 Limitations of current research and future trajectories

This review has several limitations. As a narrative synthesis, it did not perform formal risk-of-bias assessment, quality grading, or statistical meta-analysis of primary studies. The database search was executed and locked in May 2026, and articles published after this index date were not screened. Although no language restrictions were imposed in the initial search, only English-language full-text articles were finally included, potentially excluding relevant non-English publications. Additionally, the rapid evolution of the field means that ongoing Phase II/III trials of microbiome-targeted interventions may soon update or supersede some of the preclinical conclusions presented here.

## Glossary

3-IAld: 3-Indolealdehyde
AHR: Aryl hydrocarbon receptor
Akk: Akkermansia muciniphila
APCs: Antigen-presenting cells
CAFs: Cancer-associated fibroblasts
c-di-AMP: Cyclic di-adenosine monophosphate
cGAMP: Cyclic GMP-AMP
cGAS: Cyclic GMP-AMP synthase
CTLA-4: Cytotoxic T-lymphocyte-associated antigen-4
DCs: Dendritic cells
FMT: Fecal microbiota transplantation
GSDME: Gasdermin E
HDAC: Histone deacetylase
ICIs: Immune checkpoint inhibitors
IFN: Interferon
IFN-γ: Interferon-gamma
IL: Interleukin
ILA: Indole-3-lactic acid
IPA: Indole-3-propionic acid
irAEs: Immune-related adverse events
LGG: Lactobacillus rhamnosus GG
NK: Natural killer (cells)
NKT: Natural killer T (cells)
NOD2: Nucleotide-binding oligomerization domain-containing protein 2
NSCLC: Non-small cell lung cancer
OS: Overall survival
PD-L1: Programmed death-ligand 1
PD-L2: Programmed death-ligand 2
PERK: Protein kinase RNA-like endoplasmic reticulum kinase
PFS: Progression-free survival
PI3K: Phosphoinositide 3-kinase
PPARα: Peroxisome proliferator-activated receptor alpha
SCFAs: Short-chain fatty acids
SHP2: SH2-containing protein tyrosine phosphatase 2
STING: Stimulator of interferon genes
TILs: Tumor-infiltrating lymphocytes
TMAO: Trimethylamine N-oxide
TMB: Tumor mutational burden
TME: Tumor microenvironment
TNF-α: Tumor necrosis factor-alpha
Tregs: Regulatory T cells.
