# Supplementary material for: Navigating the gut–metabolite–immune axis: enhancing efficacy and mitigating toxicity of immune checkpoint inhibitors
Source: Front Immunol. 2026 Jul 13;17:1803970. doi: 10.3389/fimmu.2026.1803970 (PMC13402374; doi:10.3389/fimmu.2026.1803970)
Supplement: Supplementary file 1 [file DataSheet1.pdf]

Search Strategy

Search Date: May 1, 2026
Databases: PubMed/MEDLINE (via NCBI), Web of Science Core Collection, Embase (via Elsevier)
Search Conducted by: Z.Y. and W.S.
Review Type: Narrative review with structured literature search

1. PubMed/MEDLINE

Step	Search Terms	Results*
#1	("Gut Microbiome"[MeSH Terms] OR "Gastrointestinal Microbiome"[MeSH Terms] OR "Intestines/microbiology"[MeSH Terms] OR "gut microbiota"[Title/Abstract] OR "intestinal microbiota"[Title/Abstract] OR "gut flora"[Title/Abstract] OR "enteric microbiome"[Title/Abstract] OR "Akkermansia"[Title/Abstract] OR "Akkermansia muciniphila"[Title/Abstract] OR "Bacteroides"[Title/Abstract] OR "Bifidobacterium"[Title/Abstract] OR "Faecalibacterium"[Title/Abstract] OR "Lactobacillus"[Title/Abstract] OR "Enterococcus"[Title/Abstract] OR "Ruminococcus"[Title/Abstract] OR "Ruminococcaceae"[Title/Abstract] OR "Roseburia"[Title/Abstract] OR "Prevotellaceae"[Title/Abstract] OR "Clostridiales"[Title/Abstract] OR "fecal microbiota transplantation"[Title/Abstract] OR "FMT"[Title/Abstract] OR "probiotics"[MeSH Terms] OR "probiotic*"[Title/Abstract] OR "prebiotics"[Title/Abstract] OR "prebiotic*"[Title/Abstract] OR "postbiotics"[Title/Abstract] OR "postbiotic*"[Title/Abstract] OR "dietary fiber"[Title/Abstract] OR "dietary fibres"[Title/Abstract] OR "short-chain fatty acids"[MeSH	660,318

	Terms] OR "short chain fatty acid*"[Title/Abstract] OR "SCFA*"[Title/Abstract] OR "butyrate*"[Title/Abstract] OR "propionate*"[Title/Abstract] OR "acetate*"[Title/Abstract] OR "indole*"[Title/Abstract] OR "tryptophan"[Title/Abstract] OR "tryptophan metabolite*"[Title/Abstract] OR "indole-3-aldehyde"[Title/Abstract] OR "3-IAld"[Title/Abstract] OR "indole-3-propionic acid"[Title/Abstract] OR "IPA"[Title/Abstract] OR "trimethylamine N-oxide"[Title/Abstract] OR "trimethylamine N oxide"[Title/Abstract] OR "TMAO"[Title/Abstract] OR "bile acid*"[Title/Abstract] OR "branched-chain amino acid*"[Title/Abstract])	
#2	("Immune Checkpoint Inhibitors"[MeSH Terms] OR "Immune Checkpoint Blockade"[Title/Abstract] OR "immune checkpoint inhibitor*"[Title/Abstract] OR "ICI"[Title/Abstract] OR "ICB"[Title/Abstract] OR "PD-1"[Title/Abstract] OR "PD-L1"[Title/Abstract] OR "programmed cell death 1"[Title/Abstract] OR "programmed death ligand 1"[Title/Abstract] OR "CTLA-4"[Title/Abstract] OR "cytotoxic T-lymphocyte-associated antigen 4"[Title/Abstract] OR "LAG-3"[Title/Abstract] OR "TIM-3"[Title/Abstract] OR "TIGIT"[Title/Abstract] OR "VISTA"[Title/Abstract] OR "CD47"[Title/Abstract] OR "SIRPα"[Title/Abstract] OR "B7-H3"[Title/Abstract] OR "ipilimumab"[Title/Abstract] OR "nivolumab"[Title/Abstract] OR "pembrolizumab"[Title/Abstract] OR "atezolizumab"[Title/Abstract] OR "relatlimab"[Title/Abstract] OR "durvalumab"[Title/Abstract] OR "avelumab"[Title/Abstract] OR "cemiplimab"[Title/Abstract] OR "dostarlimab"[Title/Abstract])	3,185

#3	("Neoplasms"[MeSH Terms] OR "Carcinoma"[MeSH Terms] OR "cancer*"[Title/Abstract] OR "neoplasm*"[Title/Abstract] OR "tumor*"[Title/Abstract] OR "tumour*"[Title/Abstract] OR "malignan*"[Title/Abstract] OR "immunotherapy"[MeSH Terms] OR "immunotherapy"[Title/Abstract] OR "therapeutic response"[Title/Abstract] OR "treatment response"[Title/Abstract] OR "clinical response"[Title/Abstract] OR "objective response"[Title/Abstract] OR "efficacy"[Title/Abstract] OR "effectiveness"[Title/Abstract] OR "outcome*"[Title/Abstract] OR "survival"[Title/Abstract] OR "resistance"[Title/Abstract] OR "refractory"[Title/Abstract] OR "immune-related adverse event*"[Title/Abstract] OR "irAE*"[Title/Abstract] OR "immune related adverse event*"[Title/Abstract] OR "toxicity"[Title/Abstract] OR "adverse effect*"[Title/Abstract] OR "colitis"[Title/Abstract] OR "enterocolitis"[Title/Abstract] OR "diarrhea"[Title/Abstract] OR "diarrhoea"[Title/Abstract] OR "myocarditis"[Title/Abstract] OR "hepatitis"[Title/Abstract] OR "pneumonitis"[Title/Abstract] OR "hypophysitis"[Title/Abstract] OR "thyroiditis"[Title/Abstract] OR "dermatitis"[Title/Abstract] OR "nephritis"[Title/Abstract] OR "arthritis"[Title/Abstract] OR "cGAS-STING"[Title/Abstract] OR "cGAS"[Title/Abstract] OR "STING"[Title/Abstract] OR "cyclic GMP-AMP synthase"[Title/Abstract] OR "NOD2"[Title/Abstract] OR "nucleotide-binding oligomerization domain-containing protein 2"[Title/Abstract] OR "AhR"[Title/Abstract] OR "aryl hydrocarbon receptor"[Title/Abstract] OR "interferon*"[Title/Abstract] OR "IFN*"[Title/Abstract] OR	12,413,434

	"cytokine*"[Title/Abstract] OR "tumor microenvironment"[Title/Abstract] OR "TME"[Title/Abstract] OR "CD8+ T cell*"[Title/Abstract] OR "cytotoxic T lymphocyte*"[Title/Abstract] OR "dendritic cell*"[Title/Abstract] OR "T-cell*"[Title/Abstract])	
#4	#1 AND #2 AND #3	2975
#5	#4 Filters: English language; from database inception to May 1, 2026	2890

2. Web of Science Core Collection

Step	Search Terms	Results*
#1	TS=("gut microbiome" OR "intestinal microbiota" OR "gut microbiota" OR "gut flora" OR "enteric microbiome" OR "Akkermansia" OR "Akkermansia muciniphila" OR "Bacteroides" OR "Bifidobacterium" OR "Faecalibacterium" OR "Lactobacillus" OR "Enterococcus" OR "Ruminococcus" OR "Ruminococcaceae" OR "Roseburia" OR "Prevotellaceae" OR "Clostridiales" OR "fecal microbiota transplantation" OR "FMT" OR "probiotics" OR "probiotic*" OR "prebiotics" OR "prebiotic*" OR "postbiotics" OR "postbiotic*" OR "dietary fiber" OR "dietary fibres" OR "short chain fatty acid*" OR "SCFA*" OR "butyrate*" OR "propionate*" OR "acetate*" OR "indole*" OR "tryptophan" OR "tryptophan metabolite*" OR "indole-3-aldehyde" OR "3-IAld" OR "indole-3-propionic acid" OR "IPA" OR "trimethylamine N-oxide" OR "trimethylamine N oxide" OR "TMAO" OR "bile acid*" OR "branched-chain amino acid*")	805868
#2	TS=("immune checkpoint inhibitor*" OR "immune checkpoint blockade" OR "ICI"	194863

	OR "ICB" OR "PD-1" OR "PD-L1" OR "programmed cell death 1" OR "programmed death ligand 1" OR "CTLA-4" OR "cytotoxic T-lymphocyte-associated antigen 4" OR "LAG-3" OR "TIM-3" OR "TIGIT" OR "VISTA" OR "CD47" OR "SIRPα" OR "B7-H3" OR "ipilimumab" OR "nivolumab" OR "pembrolizumab" OR "atezolizumab" OR "relatlimab" OR "durvalumab" OR "avelumab" OR "cemiplimab" OR "dostarlimab")	
#3	TS=("cancer*" OR "neoplasm*" OR "tumor*" OR "tumour*" OR "malignan*" OR "immunotherapy" OR "therapeutic response" OR "treatment response" OR "clinical response" OR "objective response" OR "efficacy" OR "effectiveness" OR "outcome*" OR "survival" OR "resistance" OR "refractory" OR "immune-related adverse event*" OR "irAE*" OR "immune related adverse event*" OR "toxicity" OR "adverse effect*" OR "colitis" OR "enterocolitis" OR "diarrhea" OR "diarrhoea" OR "myocarditis" OR "hepatitis" OR "pneumonitis" OR "hypophysitis" OR "thyroiditis" OR "dermatitis" OR "nephritis" OR "arthritis" OR "cGAS-STING" OR "cGAS" OR "STING" OR "cyclic GMP-AMP synthase" OR "NOD2" OR "nucleotide-binding oligomerization domain-containing protein 2" OR "AhR" OR "aryl hydrocarbon receptor" OR "interferon*" OR "IFN*" OR "cytokine*" OR "tumor microenvironment" OR "TME" OR "CD8+ T cell*" OR "cytotoxic T lymphocyte*" OR "dendritic cell*" OR "T-cell*")	14534628
#4	#1 AND #2 AND #3	4073
#5	#4 Refined by: Languages=(English); Timespan=All years (1900–2026)	4073

3. Embase (via Elsevier)

Step	Search Terms	Results*
#1	(gut microbi*:ti,ab OR intestinal microbi*:ti,ab OR gut flor*:ti,ab OR enteric microbi*:ti,ab OR Akkermansia:ti,ab OR Bacteroides:ti,ab OR Bifidobacterium:ti,ab OR Faecalibacterium:ti,ab OR Lactobacillus:ti,ab OR Enterococcus:ti,ab OR Ruminococcus:ti,ab OR Roseburia:ti,ab OR FMT:ti,ab OR faecal microbiota transplantation:ti,ab OR probiotic*:ti,ab OR prebiotic*:ti,ab OR postbiotic*:ti,ab OR short chain fatty acid*:ti,ab OR SCFA*:ti,ab OR butyrate*:ti,ab OR tryptophan metabolit*:ti,ab OR TMAO:ti,ab OR bile acid*:ti,ab)	86863
#2	(immune checkpoint inhibitor*:ti,ab OR ICI:ti,ab OR ICB:ti,ab OR PD-1:ti,ab OR PD-L1:ti,ab OR CTLA-4:ti,ab OR LAG-3:ti,ab OR TIM-3:ti,ab OR TIGIT:ti,ab OR ipilimumab:ti,ab OR nivolumab:ti,ab OR pembrolizumab:ti,ab OR atezolizumab:ti,ab OR durvalumab:ti,ab)	236299
#3	(cancer*:ti,ab OR neoplasm*:ti,ab OR tumo*:ti,ab OR malignan*:ti,ab)	6177862
#4	#1 AND #2 AND #3	345
#5	#4 AND [english]/lim AND [1900-2026]/py	345

Abbreviations: [MeSH Terms], Medical Subject Headings; TS, Topic search; ti,ab, title and abstract; /de, Emtree controlled vocabulary; *, truncation; " ", phrase search.
